# Characterization of novel *CASQ1* variants in two families with unusual phenotypic features

**DOI:** 10.1007/s00415-025-13512-3

**Published:** 2025-11-28

**Authors:** Milla Laarne, Manu Jokela, Fang Zhao, Sanna Huovinen, Cornelia Kornblum, Jens Reimann, Mridul Johari, Anna Vihola, Jaakko Sarparanta, Bjarne Udd, Peter Hackman, Vilma-Lotta Lehtokari, Katarina Pelin

**Affiliations:** 1 Folkhälsan Research Center, Biomedicum Helsinki, Haartmaninkatu 8, 00290 Helsinki, Finland; 2 Department of Medical Genetics, Medicum, University of Helsinki, Helsinki, Finland; 3 Clinical Neurosciences, University of Turku, Turku, Finland; 4 Neuromuscular Research Center, Tampere University and Tampere University Hospital, Tampere, Finland; 5 Department of Pathology, University of Helsinki and Helsinki University Hospital, Helsinki, Finland; 6 Department of Pathology, Fimlab Laboratories, Tampere University Hospital, Tampere, Finland; 7 Department of Neuromuscular Diseases, Center for Neurology, University Hospital Bonn, Bonn, Germany; 8 Harry Perkins Institute of Medical Research, Centre for Medical Research, University of Western Australia, Nedlands, WA Australia; 9 Laboratory of Genetics, HUS Diagnostic Center, University of Helsinki and Helsinki University Hospital, Helsinki, Finland; 10 Molecular and Integrative Biosciences Research Programme, Faculty of Biological and Environmental Sciences, University of Helsinki, Helsinki, Finland; 11 Neurocenter, Turku University Hospital, Turku, Finland

**Keywords:** Myopathy, Calsequestrin-1, CASQ1, SR-feet

## Abstract

**Background:**

Variants in *CASQ1*, encoding a calcium-binding protein in the fast-twitch fibers of skeletal muscle, cause sarcoplasmic reticulum aberrations such as large vacuoles with CASQ1 inclusions or, less commonly, tubular aggregates. To date, seven pathogenic variants have been described, all dominant missense variants. The typical symptoms of the disease include muscle weakness, cramps, myalgia, and fatigue.

**Methods:**

We used genome and exome sequencing to identify the disease-causing variants in two families with dominant myopathy. The candidate variants were further characterized by cell-transfection studies and western blotting.

**Results:**

In Family 1, three patients presented with exercise intolerance, cramps, and myalgia. Additionally, the proband had muscle weakness and her muscle biopsy showed nemaline bodies. In electron microscopy, there were morphological changes in the triads and the SR-feet in all patients. A variant in *CASQ1*, p.(Glu89Lys), was found in all patients, whereas the proband had also two compound heterozygous variants in *NEB*. In Family 2, three patients presented with progressive muscle weakness. The proband’s muscle biopsy showed marked atrophy. The frameshift variant p.(Gly383Alafs*39) in *CASQ1* was found in all three patients. In silico analysis indicated that the variant results in protein extension, which was confirmed by western blotting of patient muscle. Cell-transfection studies showed that the variant protein forms aggregates.

**Conclusion:**

This study expands the spectrum of pathogenic *CASQ1* variants. The morphological changes in the SR-feet indicate a novel pathogenetic mechanism.

## Introduction

Variants in *CASQ1* (MIM ID *114250, cytogenetic location 1q23.2) are a known cause of vacuolar myopathy with CASQ1 aggregates (MIM ID #616231). Thus, the disease belongs to the class of protein aggregate myopathies, which are characterized by the aggregation of proteins within muscle fibers [1]. Tomelleri et al*.* [2] described the first patients with CASQ1 aggregate myopathy, and since then seven variants have been linked to the disease: p.(Asp244Gly) [3–7], p.(Asp44Asn) [8], p.(Asn56Tyr) [9], p.(Gly103Asp) [6, 8, 9], p.(Ile385Thr) [8], p.(Val256Met) [10], and p.(Asp244His) [11]. The disease onset is in adulthood and the course of disease is either nonprogressive or slowly progressive, typical symptoms being proximal and/or distal muscle weakness, myalgia, exercise intolerance, cramps, and fatigue [6]. A characteristic feature is the presence of either tubular aggregates or optically empty vacuoles in muscle, both of which stain positively for sarcoplasmic reticulum (SR) proteins [6]. In some cases, the only manifestation of the disease is elevated plasma creatine kinase (hyperCKaemia) [3, 7].

The product of *CASQ1*, calsequestrin-1, is a calcium-binding protein with high expression in the fast twitch fibers of skeletal muscle [12]. Another isoform, calsequestrin-2 (*CASQ2*), is expressed together with *CASQ1* in the slow twitch fibers and in smooth muscle, whereas cardiomyocytes express only *CASQ2* [13–15]. Due to its high binding capacity and low affinity for Ca^2+^, CASQ1 is the most crucial Ca^2+^ buffering protein in the SR [16]. It acts as a sensor for luminal Ca^2+^ concentration and regulates Ca^2+^ release through the ryanodine receptor 1 (RyR1) Ca^2+^ channels [17, 18]. In addition, CASQ1 associates with STIM1 to negatively regulate store-operated calcium entry [19, 20].

The Ca^2+^ buffering ability of CASQ1 is dependent on dynamic, Ca^2+^-induced conformational changes [16]. Ca^2+^ binding triggers the formation of CASQ1 polymers, which in turn bind increasing amounts of Ca^2+^ since the polymers offer a vast acidic surface for the ions to adsorb [21]. The consecutive aspartate stretch (CAS) domain, found at the C-terminus of the protein and consisting of eight aspartic acid residues, first saturates with Ca^2+^ before the ions spread to cover the rest of the protein surface [22]. Polymerization begins with the formation of dimers via front-to-front interaction of two monomers, and the resulting dimers then stack back to back, forming linear polymers [21, 23]. Ca^2+^ binding neutralizes the acidic CAS domain, allowing for the back-to-back stacking to occur [22, 24]. Conversely, when the SR is depleted of Ca^2+^, CASQ1 depolymerizes [25]. Due to the presence of higher- and lower-affinity Ca^2+^ binding sites, however, complete depolymerization is not necessary for Ca^2+^ to be released from the SR [22, 25].

One of the most common congenital myopathies is nemaline myopathy (NM) [26]. A characteristic feature of NM is the presence of nemaline bodies, which are aggregates of Z-disc and thin filament proteins, in Gömöri trichrome-stained muscle biopsy sections [27]. Variants in *NEB* (MIM ID*161650, cytogenetic location 2q23.3) are a frequent cause of NM and related disorders [28, 29]. The phenotype of *NEB*-NM ranges from mild to severe congenital myopathy [28].

The term “double trouble” refers to a dual genetic diagnosis, i.e., a situation where an individual has inherited two pathogenic variants in two different genes. Both variants contribute to the phenotype, which complicates the diagnostic process. As monogenic Mendelian diseases are rare, double trouble is even rarer; in one retrospective analysis with close to 7400 exome-sequenced patients, 4.9% were diagnosed with two or more molecular diagnoses, while the overall diagnostic yield was 28.2% [30]. These complex cases may also be underdiagnosed.

Here, we describe two novel *CASQ1* variants in two families with a dominant myopathy: NM_001231.5:c.265G > A p.(Glu89Lys) in Family 1 and NM_001231.5:c.1148del p.(Gly383Alafs*39) in Family 2. The proband of Family 1 was also compound heterozygous for two recessive variants in *NEB*, resulting in a concomitant nemaline myopathy. In Family 1, the symptoms included cramps, exercise intolerance, and myalgia. As a new feature of *CASQ1*-related myopathy, there were morphological changes in the SR-feet. In Family 2, the disease manifested as muscle weakness and severe fatty atrophy of skeletal muscle.

## Materials and methods

### Patients

The patients underwent a thorough clinical examination including muscle magnetic resonance imaging (MRI) as well as histopathological and molecular genetic studies to identify the causative genetic variants.

### Morphological studies of muscle biopsies

II-2, III-2, and IV-1 of Family 1 underwent a muscle biopsy from the tibialis anterior muscle at the age of 67, 45, and 20 years, respectively. Sections were stained with Gömöri trichrome and NADH-TR. Immunostaining for CASQ1 (anti-calsequestrin 2 + calsequestrin 1 antibody, ab3516, Abcam, Cambridge, UK; RRID: AB_303865) diluted 1:100, SERCA1 (anti-SERCA1 ATPase antibody VE121G9, ab2819, Abcam; RRID: AB_2061279) diluted 1:6000, SERCA2 (anti-SERCA2 antibody IID8, MAB2636, Sigma–Aldrich, St. Louis, MO, USA; RRID: AB_10615780) diluted 1:2000, RyR1 (anti-ryanodine receptor antibody 34 C, ab2868, Abcam; RRID: AB_2183051) diluted 1:1000, and DHPR (CaV1.1 monoclonal antibody 1 A, MA3-920, Thermo Fisher Scientific, Waltham, MA, USA; RRID: AB_2069575) diluted 1:600 was also performed. A muscle biopsy from vastus lateralis was obtained from the proband of Family 2 (II-1) at the age of 60 years. Sections were stained with Gömöri trichrome and hematoxylin–eosin staining.

### Electron microscopy

Immediately after biopsy, the muscle samples were fixed in 2.5% glutaraldehyde for 1 h and then post-fixed in 1% osmium tetroxide for another 1 h. The samples were embedded in the epoxy resin LX112 (Ladd Research Industries Inc., Williston, VT, USA). Sections of 60–80 nm thickness were cut and double stained with uranyl acetate and lead citrate. The sections were observed under the JEM-1400 transmission electron microscope (Jeol Ltd., Tokyo, Japan) coupled with the Morada camera (EMSIS GmbH, Münster, Germany).

### Genetic analyses

The proband of Family 1 was analyzed by whole-genome sequencing at Nebula genomics (San Francisco, CA, USA). The segregation of the *CASQ1* and *NEB* variants in the family members was determined by Sanger sequencing.

The proband of Family 2 was analyzed with the neuromuscular gene panel MyoCap version 5 [31]. In addition, whole-exome sequencing was performed at Blueprint Genetics (Helsinki, Finland) for the three affected family members, and the segregation of the *CASQ1* variant was confirmed by Sanger sequencing. Primer sequences and PCR conditions are available upon request.

### In silico investigations and variant classification

The protein sequence of human CASQ1 was retrieved from Uniprot (accession number P31415) [32]. Orthologous sequences of mouse (*Mus musculus*), rat (*Rattus norvegicus*), sheep (*Ovis aries*), chimpanzee (*Pan troglodytes*), cattle (*Bos taurus*), and pig (*Sus scrofa*) CASQ1 were retrieved from the OMA Browser [33]. The sequences were aligned and visualized with JalviewJS Test 2.11.3.3 [34]. Pathogenicity of the missense variants was assessed with the following predictors: REVEL [35], CADD [36], MetaRNN [37], AlphaMissense [38], and MutationTaster2021 [39]. The predicted effect of p.(Gly383Alafs*39) on the protein sequence was retrieved from Mutalyzer 3.0.7 [40]. The variants were classified according to the American College of Medical Genetics and Genomics (ACMG) variant classification guidelines [41] and the ClinGen Sequence Variant Interpretation Working Group recommendations [42].

### Generation of CASQ1 constructs

pUC57 constructs carrying the CASQ1 wild-type and c.1148del sequences were purchased from GenScript Inc. (Piscataway, NJ, USA). For transient expression in mammalian cells, the inserts were transferred to pCDNA5/TO vectors using standard restriction cloning methods. A construct carrying the c.265G > A variant was created from the wild-type construct by means of site-directed mutagenesis using the NEBuilder HiFi DNA Assembly Cloning Kit (New England Biolabs, Ipswich, MA USA).

### Cell culture and transient DNA transfection

HeLa cells (CCL-2, ATCC, Manassas, VA, USA; RRID:CVCL_0030) were grown in minimum essential medium (MEM, Thermo Fisher Scientific) supplemented with 10% fetal bovine serum (FBS) (Thermo Fisher Scientific), 1% GlutaMax (Thermo Fisher Scientific), and 1% penicillin/streptomycin (Thermo Fisher Scientific). The cells were transfected with the pCDNA5/TO-CASQ1 constructs using the FuGENE 6 Transfection Reagent (Promega, Madison, WI, USA) according to the manufacturer’s instructions. Expression time was 48 h.

### Thapsigargin treatment and immunofluorescence analysis

Transfected HeLa cells were treated with 3 µM thapsigargin (Sigma–Aldrich) or DMSO for 30 min at 37 °C and subsequently fixed for 10 min with 4% paraformaldehyde, permeabilized for 10 min with 0.2% Triton-X100, and blocked for 30 min with 5% BSA in PBS. The cells were incubated overnight at 4 °C with mouse monoclonal anti-calsequestrin-1 antibody (MA3-913, Thermo Fisher Scientific, RRID:AB_325496), diluted 1:100 in 1% BSA in PBS, and for 60 min at room temperature with Alexa Fluor Plus 594 Donkey anti-mouse IgG (A21203, Thermo Fisher Scientific, RRID: AB_2535789) diluted 1:500 in 1% BSA in PBS. Nuclei were stained with Hoechst, diluted 1:5000 in PBS, for 5 min at room temperature. The cells were examined using the Axio Imager M2 microscope with the Zeiss AxioCam 503 system (software Zeiss Zen 2.3 Blue, Carl Zeiss Microscopy GmbH, Germany, RRID:SCR_013672).

### Western blot

Muscle biopsy of the Family 2 proband (II-1) as well as a control muscle biopsy was homogenized in sample buffer containing 0.5 M Tris–HCl pH 6.8, 4% SDS, 8% glycerol, 10% β-mercaptoethanol, and bromophenol blue. The homogenate was heated at 95 °C for 5 min and centrifuged at 13,000 rpm for 5 min. Proteins were separated in 4–15% Mini-PROTEAN® TGX™ Precast Protein Gels (Bio-Rad Laboratories, Hercules, CA, USA), and transferred on a nitrocellulose membrane using the Trans-Blot Turbo system (Bio-Rad). Total protein was stained with the Revert 520 Total Protein Stain (Li-Cor Biosciences, Lincoln, NE, USA). The membrane was probed for CASQ1 using mouse monoclonal anti-calsequestrin-1 antibody (MA3-913) at a 1:2000 dilution at + 4 °C overnight. Alexa Fluor Plus 800 donkey anti-mouse IgG (A32789, Thermo Fisher Scientific, RRID:AB_2762832), diluted 1:10,000, was used as the secondary antibody. The fluorescent signal was detected with the Odyssey M Imaging System (Li-Cor, RRID:SCR_025709).

## Results

### Clinical description

#### Family 1

The proband (III-2) reported having been clumsy as a child but able to do sports to some extent. Since the age of 31 years, she started to experience muscle fatiguability. Currently, walking a few kilometers causes myalgia in her thighs, buttocks, and calves. During the last few years, she has had dysphagia with dry foods. She reports muscle cramps in calves and abdominal muscles, as well as widespread muscle twitching. She is able to climb one flight of stairs. The CK value has always been within the normal range.

Neurological examination at the age of 46 years showed mild proximal lower limb weakness. Getting up from a squat without using hands was difficult. Toe and heel walking was normal. There was MRC 4/5 weakness of deep finger flexors, but otherwise no obvious limb or axial muscle weakness or atrophy. No spontaneous activity or percussion abnormality of muscles was detected. Pupils were miotic and poorly reactive to light. There were no tendon contractures. Lower limb magnetic resonance imaging (MRI) showed severe fatty replacement in the semitendinosus muscles bilaterally and less extensively in the anterior compartment muscles of the lower legs.

The proband’s mother (II-2) reported always having been poor at sports and never been able to run more than short distances. She was able to do sedentary office work until normal retirement age. She is currently able to walk a few kilometers with rest pauses. She has a long-standing history of daily muscle cramps in her hands, feet, lower legs, and abdominal muscles.

Neurological examination at the age of 67 years showed mild proximal weakness. She was unable to get up from the squat without using hands. Toe and heel walking was normal. Pupils were miotic and poorly reactive to light. There was mild atrophy in the distal quadriceps muscles. Otherwise, manual muscle testing of limb and axial muscles was normal and there was no muscular atrophy or abnormal spontaneous activity. Lower limb MRI showed minor distal fatty replacement in the quadriceps muscles.

The proband’s daughter (IV-1) was able to play basketball until she was 15 years old. Thereafter, she has had myalgias affecting the thenar muscles, upper arms, thighs, and calves. Exercise capacity has reduced and the patient has been unable to run since the age of 17–18 years. Walking reportedly becomes stiff and slow after about 5–10 min. After walking for longer periods of time, she has myalgias for several days.

Neurological examination at age 20 years did not show any focal muscle weakness, atrophy, or spontaneous activity. She was able to do a squat and walk on her toes and heels. Pupil size and light reactions were normal. Lower limb MRI was normal.

Furthermore, the proband’s grandfather (I-1) was reportedly affected with similar symptoms, such as cramps and myalgia.

#### Family 2

The proband (II-1) developed muscle weakness at the age of 53 years, which started with the foot flexion and later spread to proximal lower extremities and hands. His medical history included scoliosis and a form of torticollis with partial correction by surgery. The patient later developed atrial fibrillation. CK was mildly elevated to 2.3 × ULN.

His younger sister (II-4) was known to have distal weakness and myotonic EMG. The sister’s daughter (III-1) presented without subjective symptoms, but upon clinical examination at the age of 31 years showed mild weakness of foot extension and the hip girdle muscles. Further, the father of II-1 and II-4 reportedly had weak hands and feet, as did one of the father’s brothers.

Initial examination of II-1 showed loss of deep tendon reflexes at the lower extremities and atrophic weakness of the lower arms, hands, and lower extremities. There was severe weakness of finger extension (MRC 1–2), in particular of the first and second finger, but without finger flexion weakness. He also presented with weakness of the wrist extension, foot flexion (MRC 2), and hip extension (MRC 2–3), with lesser weakness of the residual lower extremity muscles. Knee extensor weakness progressed over the years and became the cause of falls and the use of a walker. Vital capacity was normal.

Neurographic studies indicated mixed sensorimotor polyneuropathy. EMG of the vastus lateralis muscle showed spontaneous activity, myotonic discharges, and myogenic CMAP values. Muscle MRI at the age of 66 years showed symmetric, almost complete fatty atrophy of the gluteal muscles and the quadriceps femoris muscle group. Adductors and the long head of biceps femoris muscle were preserved, but showed mild edema. There were few preserved muscle fiber bundles in the semitendinosus muscles. The gastrocnemii showed complete fatty atrophy, while the soleus muscles were preserved in the cranial part. The tibialis posterior muscle was mostly preserved and showed some edema. Likewise, the leg extensors and peroneal muscles were preserved only in their cranial parts.

Genetic analysis for myotonic dystrophy type I was negative (the affected sister had been tested negative for type I and type II), likewise investigations for *DES*, *FHL1*, *BAG3*, *FLNC*, *ZASP*, *CRYAB*, and *MYOT*.

### Muscle histopathology and electron microscopy

#### Family 1

**Fig. 1:**
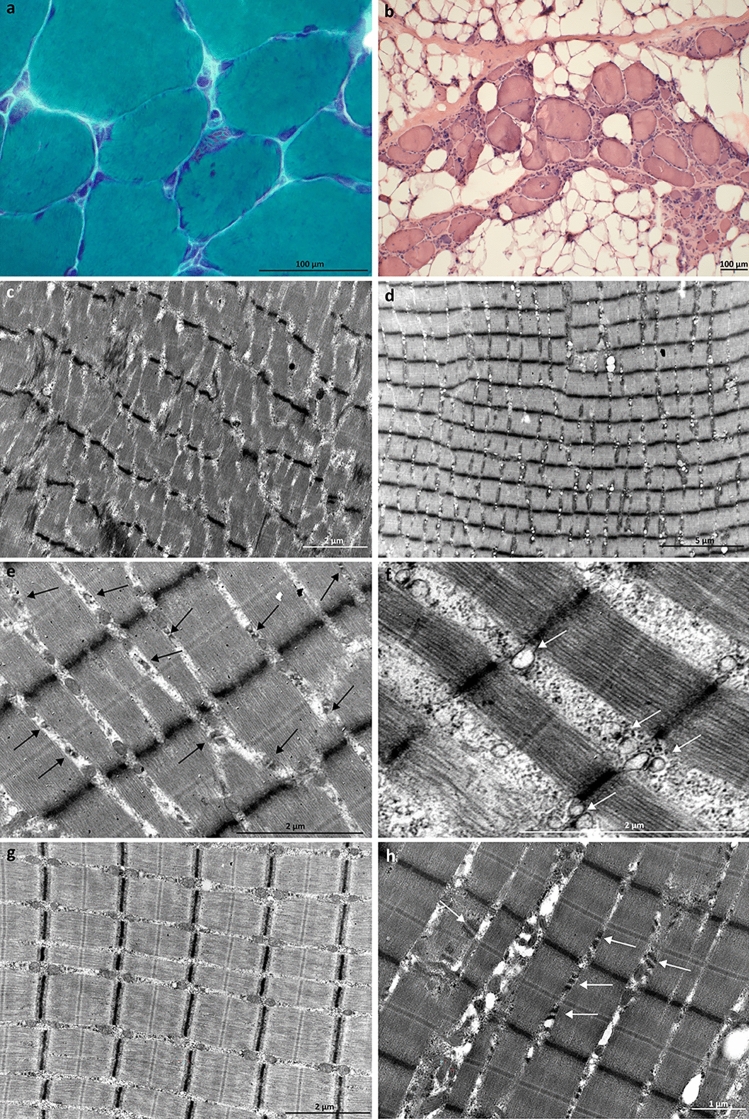
Histological and EM findings. **a** Gömöri trichrome staining of the proband of Family 1 showed nemaline bodies. **b** Hematoxylin–eosin staining of the Family 2 proband revealed marked fibrosis and atrophy, as well as rimmed vacuoles and internal nuclei. **c–h** Family 1 EM findings. **c** Mild degenerative myopathic changes, including streamed Z-lines and disorganized myofibrils, were seen in EM. **d** The triads were prominent even at low magnification. **e** The density of the SR-feet was increased. The arrows indicate the triads. **f** The TC lumen (indicated by arrows) was dilated and filled with electron-dense material. **g** Triads of normal control muscle. **h** The SR-feet (indicated by the arrows) appeared thickened, layered, and lengthened

In light microscopy, atrophic rounded or angular type 1 fibers and atrophic rounded type 2 fibers, including highly atrophic minifibers, were observed in the muscle biopsy of the proband (III-2). Gömöri trichrome staining revealed nemaline bodies in cap-like structures in some atrophic fibers, as well as rare intermyofibrillar nemaline bodies (Fig. 1a). Oxidative enzyme staining showed moth-eaten fibers or multiminicore-like changes. In the muscle biopsy of II-2, a few atrophic angular fibers in both fiber types and atrophic type 2 minifibers were observed. Oxidative enzyme staining showed again moth-eaten fibers or multiminicore-like changes. No significant myopathic pathology was observed in the muscle biopsy of IV-1 in light microscopy. Immunostaining for SR proteins—including CASQ1, SERCA1/2, and RyR1—as well as the T-tubule marker DHPR, demonstrated normal distribution in all muscle biopsies.

In electron microscopy (EM), the muscle ultrastructure was largely preserved with only mild degenerative myopathic changes, such as disorganized myofibrils and streamed Z-lines in III-2 (Fig. 1c). Besides mild general myopathic changes, alterations were found in the triads in all patients. The triads were prominent even at low magnification (8000×) (Fig. 1d). The terminal cisternae (TC) lumen was filled with electron-dense material (Fig. 1e), and the lumen was dilated (Fig. 1f) (the average of 20 measurements being 174 nm in the youngest patient IV-1 and 242 nm in the oldest patient II-2, compared to the average of 115 nm in normal muscle (Fig. 1g)). Further, proliferation of the SR-feet was noted, with high electron density, thickening, increased length, and duplication of the feet with layers (Fig. 1e and h).

#### Family 2

Vastus lateralis muscle biopsy of the proband (II-1) showed a dystrophic pattern with marked fibrosis and atrophy (Fig. 1b). Some rimmed vacuoles and few large sarcoplasmic bodies were found. There were no signs of inflammation.

### Genetic findings

#### Family 1

**Fig. 2:**
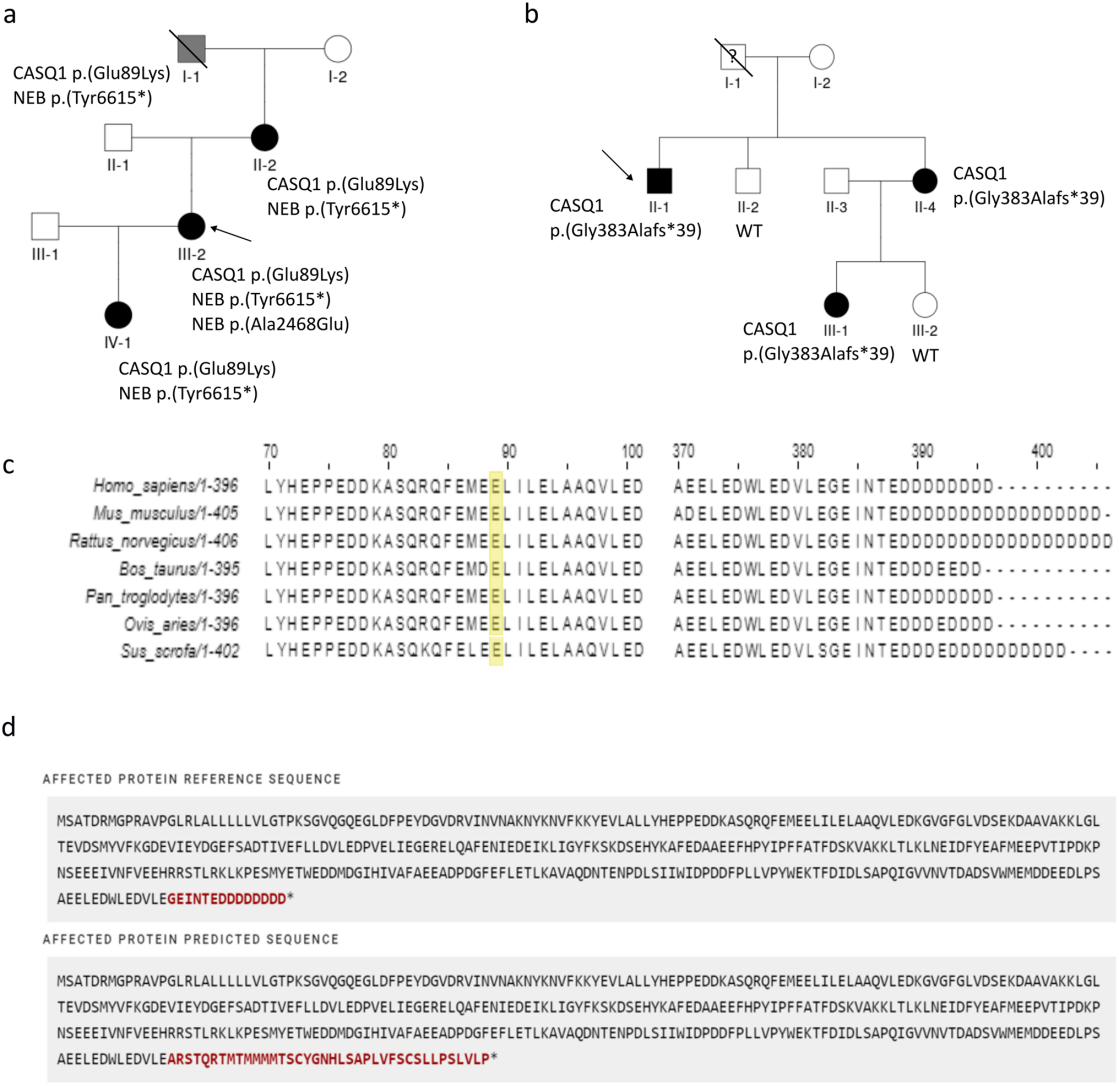
Variant segregation, conservation, and the effect of p.(Gly383Alafs*39) on the protein. **a** and **b** Segregation of the variants in the families. **a** Four affected members of Family 1 carried the p.(Glu89Lys) variant in CASQ1 and the p.(Tyr6615*) variant in NEB. The proband also had the NEB missense variant p.(Ala2468Glu) in trans with the nonsense variant. I-1 was reportedly affected, but he was not available for medical examination. **b** Three affected members of Family 2 carried the p.(Gly383Alafs*39) variant in CASQ1, which was not found in the two unaffected relatives II-2 and III-2. I-1 was not available for medical examination or genetic analysis, but he was said to have had weakness of the hands and feet. **c** Conservation of Glu89 (marked in yellow) and the C-terminal tail among mammals. **d** Predicted effect of p.(Gly383Alafs*39) on the protein (retrieved from Mutalyzer.nl). Fourteen C-terminal residues, including the conserved aspartic acid tail, are replaced, and the C-terminus is extended beyond the wild type stop codon by 24 residues

Whole-genome sequencing (WGS) analysis identified a heterozygous missense variant in exon 1 of *CASQ1* (NM_001231.5:c.265G > A p.(Glu89Lys)) in Family 1, which segregated with the disease (Fig. 2a). Segregation was confirmed by Sanger sequencing. The variant is not present in the gnomAD 4.0 database.

Furthermore, a heterozygous nonsense variant in exon 129 of *NEB* (NM_001271208.2:c.19845C > A p.(Tyr6615*)) was found in all affected individuals. The variant is not found in the gnomAD 4.0 database. In addition, the proband (III-2) carried a rare missense variant on exon 54 of *NEB* (NM_001271208.2:c.7403C > A p.(Ala2468Glu)) in trans with the nonsense variant (gnomAD Exomes 4.0: 0.000000684).

#### Family 2

Targeted sequencing with the neuromuscular gene panel MyoCap identified a one-base deletion leading to a frameshift in the last exon of *CASQ1* (NM_001231.5:c.1148del p.(Gly383Alafs*39)) in the proband (II-1) of Family 2. Exome sequencing of II-1, his sister (II-4), and the sister’s daughter (III-1) revealed no other candidate variants. Sanger sequencing confirmed that the *CASQ1* variant segregated with the disease in the family (Fig. 2b). The variant is not found in the gnomAD 4.0 database.

### In silico investigations and variant classification

The substitution of Glu89 with lysine was predicted pathogenic by many in silico predictors, e.g., REVEL 0.69, CADD 26.4, MetaRNN 0.8157, AlphaMissense 0.6221, and MutationTaster deleterious. Analysis of orthologous sequences showed that the residue is conserved across mammals (Fig. 2c). According to the guidelines of the American College of Medical Genetics and Genomics (ACMG), p.(Glu89Lys) was classified as a variant of uncertain significance (VUS; PM2: absent from population databases, PP3: computational evidence supports a deleterious effect, PP1: co-segregation with disease in multiple affected family members). The variant has been reported in ClinVar as a VUS (1 submission).

The frameshift variant p.(Gly383Alafs*39) is expected to replace 14 C-terminal residues, including the stretch of aspartic acids, and extend the C-terminus beyond the wild-type stop codon by 24 residues (Fig. 2d). The C-terminal aspartic acids are conserved among mammals (Fig. 2c), and their replacement was predicted deleterious by MutationTaster. In accordance with the ACMG variant classification guidelines and the ClinGen Sequence Variant Interpretation Working Group recommendations, the variant was classified as pathogenic (PVS1_Strong: frameshift variant not undergoing NMD and altering a domain critical to protein function, PS3: functional studies show a deleterious effect, PM2: absent from population databases, PP1: co-segregation with disease in multiple affected family members). The variant has been reported in ClinVar as a VUS (2 submissions).

The nonsense variant p.(Tyr6615*) in *NEB*, found in all affected individuals of Family 1, was classified as likely pathogenic according to the ACMG guidelines (PVS1: predicted null variant in a gene where LOF is a known mechanism of disease, PM2: absent in population databases). The p.(Ala2468Glu) variant in *NEB,* identified in the proband (II-2), was predicted pathogenic (REVEL 0.654, MetaRNN 0.9255, AlphaMissense 0.9457, MutationTaster deleterious, CADD 24.9) and was classified as a VUS (PP3: computational evidence supports a deleterious effect).

### The extended CASQ1 protein is expressed in patient muscle

**Fig. 3:**
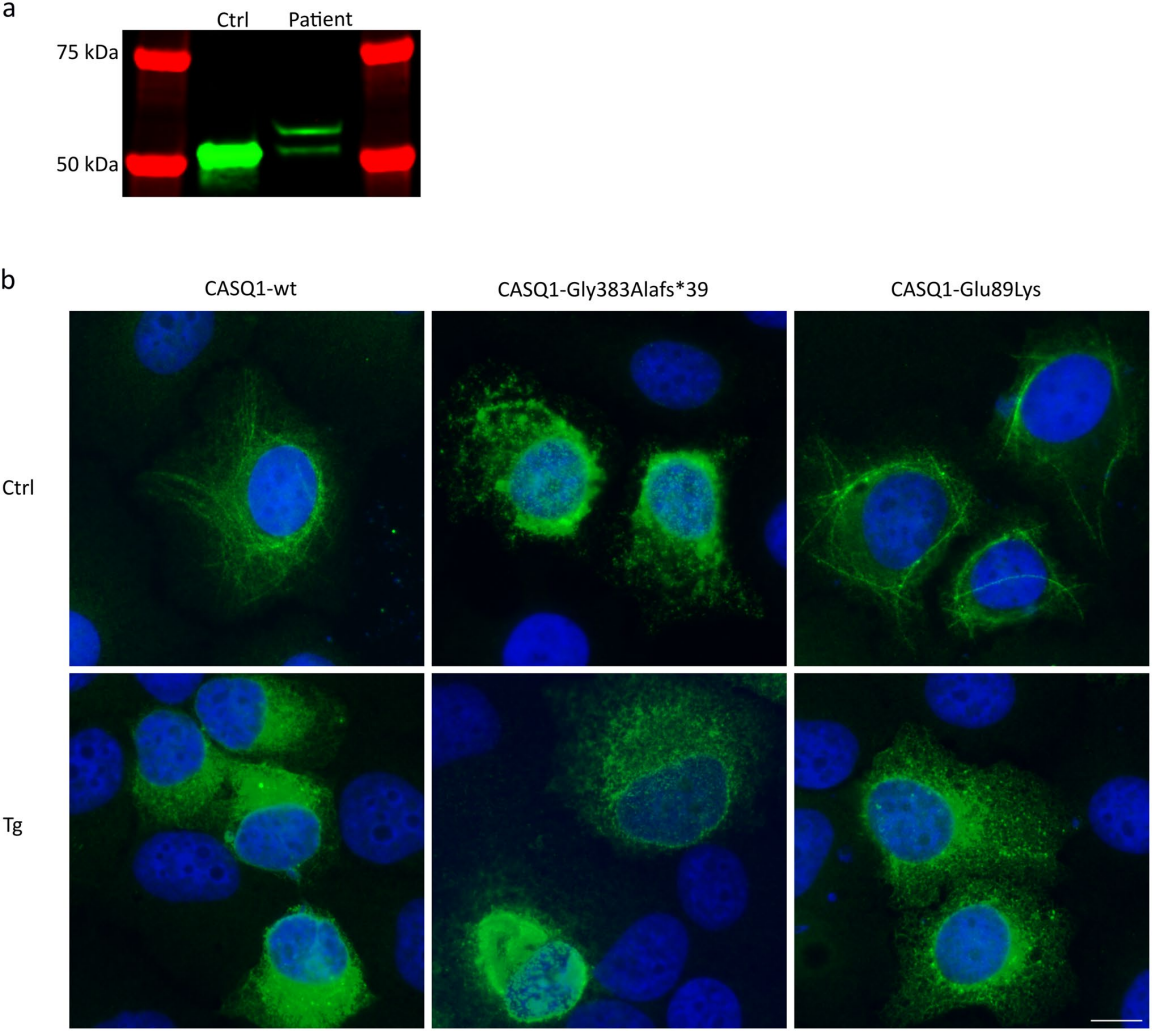
Protein-level findings. **a** Western blotting of patient muscle (Family 2, II-1) showed that the extended CASQ1 protein, caused by the frameshift variant p.(Gly383Alafs*39), is expressed on the protein level. **b** Upper panel: transfected to HeLa cells, CASQ1-wt and CASQ1-Glu89Lys polymerized, whereas CASQ1-Gly383Alafs*39 aggregated. Lower panel: when the cells were depleted of Ca^2+^ by thapsigargin treatment, CASQ1-wt and CASQ1-Glu89Lys monomerized, while CASQ1-Gly383Alafs*39 remained aggregated. Scale bar 10 µm. To visualize the polymers of CASQ1-wt-ctrl and CASQ1-Glu89Lys-ctrl, the intensity level of these images was set higher than in the other images

Expression of the extended CASQ1 protein, caused by p.(Gly383Alafs*39), in patient muscle (Family 2 proband, II-1) was analyzed by western blotting. The results showed that there was an additional band corresponding to the extended protein (Fig. 3a).

### The variant p.(Gly383Alafs*39) causes aggregation of CASQ1

Transfection of pCDNA5/TO-CASQ1-wt to HeLa cells resulted in a complex polymeric CASQ1 network, whereas CASQ1-Gly383Alafs*39 showed aggregation (Fig. 3b, upper panel). CASQ1-Glu89Lys polymerized similarly to the wild type [3, 9].

Treatment with the sarcoplasmic/endoplasmic reticulum calcium ATPase (SERCA) inhibitor thapsigargin (Tg) results in Ca^2+^ depletion from the SR, leading to monomerization of CASQ1. The effect is demonstrated with CASQ1-wt, which appeared monomeric after 30 min with 3 µM Tg (Fig. 3b, lower panel). CASQ1-Gly383Alafs*39 remained aggregated also in low-calcium conditions, while CASQ1-Glu89Lys monomerized as the wild type.

## Discussion

Here, we describe two families with a dominant myopathy carrying previously unreported variants in *CASQ1.* The phenotype was mild in Family 1, the main symptoms being fatigue, cramps, myalgia, and exercise intolerance. Such symptoms are often reported in association with *CASQ1* variants [10]. The proband’s (III-2) clinical presentation with selective fatty replacement of the semitendinosus and to a lesser extent the anterior lower leg muscles on MRI, as well as nemaline bodies on muscle biopsy, diverged from the findings of the other two family members (II-2 and IV-1). These distinctive features in the proband can be attributed to double trouble caused by the two *NEB* variants p.(Tyr6615*) and p.(Ala2468Glu). The proband’s mother (II-2) and daughter (IV-1) carried only the nonsense variant, and such variants in *NEB* are known to be recessive [28]. The mother developed mild proximal muscle weakness and minor fatty replacement in the quadriceps muscles, which may be due to longer progression of the *CASQ1*-related myopathy.

In contrast, the phenotype in Family 2 was more severe, with progressive muscle weakness and marked fatty replacement of the affected muscles. The phenotype, which did not include cramps or myalgia, was less typical for a *CASQ1* myopathy, but some cases with severe progressive weakness and muscular dystrophy as the main findings have been described previously [6, 10].

Previous reports have described vacuoles [2, 3, 7, 10] and tubular aggregates [8, 9] as pathological findings in patients with *CASQ1* mutations. However, no vacuoles or tubular aggregates were observed in the muscle biopsies of our patients, which may be due to sampling bias or end-stage pathology in the biopsied muscle. We hypothesize that in Family 1 the thickened SR-feet disturb normal excitation–contraction coupling, leading to a phenotype characterized by fatigue, cramps, and myalgia. The cellular mechanisms giving rise to the more severe dystrophic muscle pathology in Family 2 remain to be clarified.

The CAS domain, abolished by the frameshift variant p.(Gly383Alafs*39) in Family 2, is functionally important for the protein. It mediates the interactions of CASQ1 with its binding partners triadin, junctin, the RyR channels, and STIM1 [19, 43–46]. Furthermore, deletion or substitution of this domain makes CASQ1 completely or partially unable to polymerize and bind Ca^2+^ [43]. In our cell-transfection studies, CASQ1-Gly383Alafs*39 was aggregated both in the presence and absence of Ca^2+^ (Fig. 3b). Due to the replacement of muscle tissue with fibrous tissue and fat, we could not assess whether these CASQ1 aggregates were present also in the patient muscle of Family 2. It is likely, however, that the mutated protein interferes with the wild-type protein during polymerization, resulting in aggregates. Consequently, the Ca^2+^ binding capacity of the protein is likely reduced, since Ca^2+^ binding is dependent on polymerization [21]. In addition to this loss-of-function effect, it is possible that the aggregates also have a toxic gain-of-function effect within the muscle cells.

Despite its location on α-helix 2 in Domain I, which interacts with the α-helix 2 of another CASQ1 monomer in the front-to-front interface, the missense variant p.(Glu89Lys) of Family 1 did not seem to affect the polymerization ability of CASQ1 in our cell-transfection studies (Fig. 3b) [5, 23, 47]. In previous studies, turbidity assays or analytical ultracentrifugation have been used to study the aggregation propensity further [5, 8, 9]. However, as we did not see CASQ1 aggregates in any of the patient muscle biopsies analyzed, we focused on the changes in the muscle ultrastructure seen in EM (Fig. 1c–f). The SR-feet, which appeared proliferated in patient muscle (Fig. 1c–f), are also known as the RyR-feet, because they are mainly composed of the RyR1 Ca^2+^ release channels which CASQ1 regulates [17, 18, 48]. A similar effect on the RyR-feet has been seen in the muscles of CASQ1-null mice, which had several rows of RyR-feet instead of the two seen in wild-type mice [49]. The authors hypothesized that the increased number of the RyR1-channels could be a compensatory mechanism to deliver more Ca^2+^ to the contractile apparatus [49]. Additionally, in Family 1, dilation of the TC lumen and accumulation of electron-dense material inside the lumen were observed in EM. The loss of normal Ca^2+^ binding may lead to accumulation of material, such as insoluble Ca^2+^, within the TC. Widened TC lumen and tubular aggregates within the lumen are a common feature in *CASQ1*-related myopathy, but to the best of our knowledge, this is the first report of morphologically altered SR-feet [3, 6, 8, 9].

In conclusion, we report two new *CASQ1* variants in association with dominant myopathy, one of which resulted in novel alterations in the SR-feet. These findings expand the spectrum of pathogenetic *CASQ1* variants and increase our understanding of this relatively unknown disease gene.

## Data Availability

The data supporting the findings of this study are available on request from the corresponding author. The data are not publicly available due to privacy or ethical restrictions.
